# Integration of molecular breeding and multi-resistance screening for developing a promising restorer line Guihui5501 with heavy grain, good grain quality, and endurance to biotic and abiotic stresses

**DOI:** 10.3389/fpls.2024.1390603

**Published:** 2024-06-07

**Authors:** Minyi Wei, Qun Yan, Dahui Huang, Zengfeng Ma, Shen Chen, Xiaoting Yin, Chi Liu, Yuanyuan Qin, Xiaolong Zhou, Zishuai Wu, Yingping Lu, Liuhui Yan, Gang Qin, Yuexiong Zhang

**Affiliations:** ^1^ Rice Research Institute, Guangxi Academy of Agricultural Sciences/Guangxi Key Laboratory of Rice Genetics and Breeding/State Key Laboratory for Conservation and Utillzation of Subtropical Agro-bioresources, Nanning, China; ^2^ Plant Protection Research Institute, Guangxi Academy of Agricultural Sciences, Nanning, China; ^3^ Institute of Plant Protection, Guangdong Academy of Agricultural Sciences, Guangdong Provincial Key Laboratory of High Technology for Plant Protection, Guangzhou, China; ^4^ Enping Plant Protection Station, Enping, China; ^5^ Agricultural Science and Technology Information Research Institute, Guangxi Academy of Agricultural Sciences, Nanning, China; ^6^ Liuzhou Branch, Guangxi Academy of Agricultural Sciences, Liuzhou Research Center of Agricultural Sciences, Liuzhou, China

**Keywords:** rice, restorer line, marker-assisted selection (MAS), blast, brown planthopper (BPH), cold tolerance, heavy-grain, quality

## Abstract

Rice, a critical staple on a global scale, faces escalating challenges in yield preservation due to the rising prevalence of abiotic and biotic stressors, exacerbated by frequent climatic fluctuations in recent years. Moreover, the scorching climate prevalent in the rice-growing regions of South China poses obstacles to the cultivation of good-quality, heavy-grain varieties. Addressing this dilemma requires the development of resilient varieties capable of withstanding multiple stress factors. To achieve this objective, our study employed the broad-spectrum blast-resistant line Digu, the brown planthopper (BPH)-resistant line ASD7, and the heavy-grain backbone restorer lines Fuhui838 (FH838) and Shuhui527 (SH527) as parental materials for hybridization and multiple crossings. The incorporation of molecular markers facilitated the rapid pyramiding of six target genes (*Pi5*, *Pita*, *Pid2*, *Pid3*, *Bph2*, and *Wx^b^*). Through a comprehensive evaluation encompassing blast resistance, BPH resistance, cold tolerance, grain appearance, and quality, alongside agronomic trait selection, a promising restorer line, Guihui5501 (GH5501), was successfully developed. It demonstrated broad-spectrum resistance to blast, exhibiting a resistance frequency of 77.33% against 75 artificially inoculated isolates, moderate resistance to BPH (3.78 grade), strong cold tolerance during the seedling stage (1.80 grade), and characteristics of heavy grains (1,000-grain weight reaching 35.64 g) with good grain quality. The primary rice quality parameters for GH5501, with the exception of alkali spreading value, either met or exceeded the second-grade national standard for premium edible rice varieties, signifying a significant advancement in the production of good-quality heavy-grain varieties in the southern rice-growing regions. Utilizing GH5501, a hybrid combination named Nayou5501, characterized by high yield, good quality, and resistance to multiple stresses, was bred and received approval as a rice variety in Guangxi in 2021. Furthermore, genomic analysis with gene chips revealed that GH5501 possessed an additional 20 exceptional alleles, such as *NRT1.1B* for efficient nitrogen utilization, *SKC1* for salt tolerance, and *STV11* for resistance to rice stripe virus. Consequently, the restorer line GH5501 could serve as a valuable resource for the subsequent breeding of high-yielding, good-quality, and stress-tolerant hybrid rice varieties.

## 1 Introduction

Rice (*Oryza sativa* L.) stands as one of the world’s most critical food crops, experiencing significant yield improvements after two Green Revolutions (dwarfing breeding and hybrid vigor utilization). However, breeding practices for rice quality and resistance have lagged behind (Lou et al., 2023). Hybrid rice exhibits a 10%–20% yield advantage over conventional varieties (Zeng et al., 2017), profoundly enhancing China’s and the world’s food self-sufficiency. Hybrid rice breeding, a complex procedure involving the development of male-sterile lines, restorer lines, and hybrid combinations, entails lengthy breeding cycles, technical intricacies, and tedious processes. The breeding of male-sterile lines, particularly cytoplasmic male-sterile (CMS) lines, necessitates multiple rounds of selection and sterility testing to ensure their stability, which consumes significant labor and time resources. In contrast, the breeding procedure for restorer lines is more straightforward and prone to breakthroughs. Hence, efficiently consolidating high yield, good quality, and stress tolerance traits into restorer lines to develop breakthrough hybrid rice varieties represents a crucial avenue for future rice breeding research.

Blast disease and brown planthopper (BPH) infestations are two of the most severe challenges in rice production, leading to significant reductions in yield and quality deterioration (Pennisi, 2010). The rice blast disease poses a significant threat to both northern and southern rice cultivation regions in China, with Guangxi emerging as a major hotspot for this pathogen. Varying degrees of annual damage are observed across these regions due to the prevalence of the disease. In recent years, the annual occurrence of blast disease in Guangxi has reached approximately 533,000 hectares, representing roughly a quarter of the total rice cultivation area in Guangxi. Previous studies have indicated that the most economically effective and environmentally friendly approach to controlling blast disease is through the breeding of broad-spectrum and durable resistant varieties, involving the utilization of broad-spectrum resistance genes or combining multiple resistance genes (Tacconi et al., 2010; Devanna et al., 2022). To date, over 100 blast-resistant genes have been identified, with 38 R genes cloned (Ning et al., 2020). These findings provide crucial genetic resources for molecular breeding aimed at enhancing blast resistance.

The BPH is also a major pest affecting rice production (Du et al., 2009). Currently, 70 BPH resistance genes/quantitative trait loci (QTLs) have been identified in rice, with 17 resistance genes already cloned (Yan et al., 2023). Out of these, nine genes primarily utilized in BPH resistance breeding are *Bph1*, *Bph2*, *Bph3*, *Bph6*, *Bph9*, *Bph14*, *Bph15*, *Bph18*, and *Bph26*. Varieties bred with *Bph1*, *Bph2*, and *Bph3* have been widely promoted in Southeast Asian countries, showcasing the broad application of these resistance genes.

Rice, originating from tropical and subtropical regions, is a temperature-sensitive crop (Andaya and Mackill, 2003). Exposure to low temperatures during rice sowing and transplanting can lead to slow seedling growth, seedling decay, and seedling death, resulting in reduced yields. Seedling-stage cold damage is a significant concern affecting production in the double-season indica rice regions of southern China and the middle-lower reaches of the Yangtze River, where chill weather frequently returns in later spring (March–April), a phenomenon referred to as “cold spell in later spring”. In the past 15 years, Guangxi has experienced six such severe events in 2007, 2008, 2010, 2014, 2020, and 2023, causing extensive seedling decay and death, consequently resulting in significant yield losses for the early rice season. Therefore, breeding new rice varieties with strong cold tolerance during the seedling stage holds great significance for ensuring early rice production in southern China and the middle-lower reaches of the Yangtze River. It also helps prevent cold weather damage for early-seeded varieties in expanded planting regions with mid- and late-maturing varieties. Rice tolerance to low-temperature stress is a complex quantitative trait controlled by multiple genes. Currently, only a few genes related to seedling-stage cold tolerance have been identified, such as LTG1 (Lu et al., 2014), *Cold1* (Ma et al., 2015), *qCST-9* (Zhao et al., 2017), *HAN1* (Mao et al., 2019), and *OsSEH1* (Gu et al., 2023). Moreover, there are few reports on molecular breeding for improving rice seedling-stage cold tolerance.

As people’s living standards rise rapidly, Chinese consumers are increasingly demanding high-quality rice. The breeding of inbred varieties with high-quality rice has been quite successful in the past decade, as the majority of current inbred varieties produce rice of high quality that meets the first or second standard of premium-quality rice in the Chinese market. However, there is still considerable room for improvement in the quality of hybrid rice (Yang et al., 2020; Seck et al., 2023). The *Wx* locus plays a pivotal role in influencing not only amylose content (AC) and appearance quality (AQ) but also the eating and cooking quality (ECQ) of rice, thus serving as a multifunctional locus (Lou et al., 2023). Many *Wx* alleles including *wx*, *Wx* ^a^, *Wx* ^b^, *Wx* ^in^ (*Wx* ^g1^), *Wx* ^g2^, and *Wx* ^g3^ (*Wx* ^lv^) have been identified in rice (Teng et al., 2012; Huang et al., 2020). Of these *Wx* alleles, the *Wx* ^b^ allele with low amylose has been demonstrated to have significantly higher appearance, palatability, and quality phenotypes compared to other *Wx* alleles (Teng et al., 2013; Lin et al., 2023; Zhu et al., 2024). Researchers have utilized a polymorphic (CT)_n_ microsatellite located in the 5′-untranslated region of the *Wx* gene, known as molecular marker RM190, for its extensive application in assisting breeding programs. This marker has been proven instrumental in enhancing the efficiency of rice quality improvement through selective breeding (Temnykh et al., 2000; Jantaboon et al., 2011).

Grain weight is a crucial component affecting rice yield, and increasing grain weight has been proven effective in enhancing rice production (Li et al., 2016; Hao et al., 2021). However, the hot weather prevalent in the South China rice region is not conducive to the development of desirable appearance quality in heavy-grain varieties. Shuhui527 (SH527) and Fuhui838 (FH838), two breakthrough heavy-grain restorer lines in China after Minghui 63, exhibit outstanding characteristics such as superior grain quality and robust resistance to both biotic and abiotic stresses. Many hybrid rice varieties derived from these two lines demonstrate broad adaptability, strong stress resistance, and high and stable yields (Ren et al., 2021) (https://www.ricedata.cn/variety/varis/601150.htm). Notably, SH527 is one of the most frequently used restorer lines in the breeding of hybrid rice combinations in China (https://www.ricedata.cn/variety/index.htm). Both FH838 and SH527 serve as backbone parents in hybrid rice breeding, giving rise to numerous excellent restorer lines that have found extensive application in production.

In this study, FH838 and SH527, two elite heavy-grain restorer lines, ASD7, a line with intermediate resistance to BPH, and Digu, an elite germplasm for broad-spectrum resistance to rice blast, were employed as parents for hybridization and multiple crossing. Through the utilization of molecular marker-assisted selection (MAS), six target genes (*Pi5*, *Pita*, *Pid2*, *Pid3*, *Bph2*, and *Wx^b^*) were successfully pyramided. Additionally, phenotype-based selection was extensively employed to screen for blast resistance, BPH resistance, cold tolerance, grain appearance, and quality, as well as comprehensive agronomic traits. These efforts led to the successful breeding of GH5501, a superior restorer line with high-yielding potential, good grain quality, and resilience to both biotic and abiotic stresses, characterized by long and heavy grains. The development of GH5501 not only provided a valuable and groundbreaking restorer line for hybrid rice breeding programs but also exemplified a practical strategy for pyramiding high yield, good grain quality, and multiple resistances to biotic and abiotic stressors in rice breeding.

## 2 Materials and methods

### 2.1 Plant materials and growth conditions

In this study, four different materials were employed as parents for hybridization, aiming to introduce disease resistance, pest resistance, and cold tolerance into high-yielding, superior-quality, and resistant heavy-grain new lines. Specifically, ASD7 was utilized as a *Bph2* gene-containing, BPH-resistant material, while Digu served as a blast-resistant material containing *Pid1*, *Pid2*, and *Pid3* genes, which were identified in February 2004 and August 2009, respectively (Chen et al., 2004; Shang et al., 2009). Additionally, the heavy-grain restorer lines FH838 and SH527 were employed as widely used and excellent three-line hybrid rice restorer lines in China, with a thousand-grain weight of 32.90 g and 29.83 g, respectively. SH527 contains *Pi5*, *Pita*, and *Wx^b^* genes, showcasing outstanding characteristics such as excellent grain quality and resistance, while FH838 exhibits a high seed-setting rate, broad adaptability, and consistently high yields.

The control variety TY7118, part of the TY series of heavy-grain rice varieties that have dominated early rice cultivation in Guangxi for over a decade, serves as the reference variety for rice regional trials in the South of Guangxi. The three-line sterile lines used for evaluating heterosis are Nafeng A, Tianfeng A, and Longtepu A. Except for the materials employed in disease resistance identification, all other materials were cultivated in Nanning, Guangxi, situated in the southern rice region of China. Although rice was cultivated in two seasons, early and late, in this region, achieving good external grain quality posed a challenge for heavy-grain rice varieties, particularly those with early maturation.

### 2.2 Evaluation of blast disease resistance

Blast resistance evaluation of GH5501 and heavy-grain parents SH527 and FH838 was conducted collaboratively by the Plant Protection Research Institute of the Guangxi Academy of Agricultural Sciences and the Plant Protection Research Institute of the Guangdong Academy of Agricultural Sciences. This assessment involved an indoor artificial spraying test utilizing 75 *Magnaporthe oryzae* isolates collected over the past decade from various blast-prone areas in Guangxi and Guangdong. All of the materials identified underwent a spore suspension spray (1 × 10^5^ spores/mL) at the three-leaf stage in a dark environment at 26°C for 24 hours. Subsequently, they were cultured in a greenhouse with a temperature range of 24°C–30°C and a relative humidity of 90%. Seven days after inoculation, blast symptoms were evaluated using a 0–9 scale standard based on the type and size of lesions described by the International Rice Research Institute (IRRI, 2013). Rice plants showing disease rating scales of 0–3 are considered to be resistant, and those with disease rating scales of 4–9 are susceptible.

Evaluation of field blast resistance of GH5501 and NFA took place in Nansha Village, Limu Town, Cenxi City, Hezhou Base of Agricultural Science Institute of Hezhou City, and Xinrong Village, Xinjia Township, Jingxi County. These areas represent regions with the most severe natural occurrence of blast disease in Guangxi and also serve as identification points for blast disease resistance in the regional trials of rice varieties in Guangxi. The data for field blast resistance evaluation of NY5501 and TY7118 were sourced from the joint comparative and regional trials for the late-maturing rice group of early seasons in the south of Guangxi from 2018 to 2020. The resistance identification, survey methods, and grading standards adhered to the technical regulations of the Ministry of Agriculture of China, as outlined in the “Technical Regulations for Identification and Evaluation of Rice Blast Resistance in Rice Variety Trials” (NY/T2426–2014).

### 2.3 BPH resistance identification

The BPH resistance identification was delegated to the Plant Protection Research Institute of the Guangxi Academy of Agricultural Sciences. The insect source was collected from rice fields in Nanning, Guangxi. The local biotype of BPH in Nanning is a mixed biotype with strong virulence. The Standard Seedbox Screening Technique (SSST) was employed with slight modifications. Seeds were sown in metal trays, with each material in a row of approximately 25 plants, repeated three times. When seedlings reached the three-leaf stage, weak seedlings were removed, and each plant was infested with five first–second instar nymphs of BPH. After 7–10 days from the wilting of the “TN1” plants, the phenotype of each plant was evaluated according to the “R” (resistant) and “S” (susceptible) grading system outlined in the “Standard Evaluation System for Rice” (IRRI, 2013). The weighted average damage level was then calculated for each variety, where the average damage level ranged from 1.0 to 1.9 [high resistance (HR)], 2.0 to 3.9 [moderate resistance (MR)], 4.0 to 5.9 [low resistance (LR)], 6.0 to 7.9 [moderate susceptibility (MS)], and 8.0 to 9.0 [high susceptibility (HS)].

### 2.4 Cold tolerance identification methods

#### 2.4.1 Meteorological data

Natural low-temperature meteorological data were obtained from the nearby meteorological observation station of the experimental field, provided by the Nanning Meteorological Bureau. During the period from March 1 to March 30, 2014, the average temperature was only 17°C, with particularly low daily temperatures ranging from 12.5°C to 17.0°C for half a month from March 2 to March 17, representing typical low-temperature weather.

#### 2.4.2 Seedling-stage cold tolerance identification

The identification of natural low-temperature tolerance was conducted by sowing seeds on February 28, 2014, during early spring’s low-temperature weather, without covering plastic films. The assessment took place after 30 days. For artificial cold tolerance identification, seedlings were transferred to an artificial climate chamber for cold treatment upon reaching the three-leaf stage in the greenhouse. The conditions were set at a temperature of 9°C, with a relative humidity of 75% to 85%, a light intensity of 12,000 lx, with 12 hours of light and 12 hours of darkness each day, and continuous treatment for 5 days. Subsequently, the seedlings were moved to a 26°C climate chamber for recovery for 7 days, repeated three times. Following the “Standard Evaluation System for Rice” (IRRI, 2013), an assessment was conducted based on the degree of leaf redness after cold treatment. The phenotype of each seedling was observed, and the weighted average damage level was calculated for each variety. The average damage level ranged from 1.0 to 1.9 (HR), 2.0 to 3.9 (MR), 4.0 to 5.9 (LR), 6.0 to 7.9 (MS), and 8.0 to 9.0 (HS).

### 2.5 Genotyping and MAS

DNA extraction from rice fresh leaves of rice seedlings followed the simplified cetyltrimethylammonium bromide (CTAB) extraction method (Murray and Thompson, 1980). The molecular markers used in this study, as outlined in **Table 1**, were synthesized by AuGCT Biotech Co., Ltd. (Wuhan, China). PCR was primarily conducted on a PE Company 9700 PCR instrument. The reaction system (10 μL) comprised 5.0 μL of 2× Taq Plus Master Mix II, 0.5 μL each of forward and reverse primers (10 μmol/L), 1.0 μL of template DNA, and 3.0 μL of ddH_2_O. The PCR program involved an initial denaturation at 95°C for 5 min, followed by 35 cycles of denaturation at 94°C for 30 sec, annealing at 55°C for 30 sec, extension at 72°C for 40 sec, and a final extension at 72°C for 5 min. The annealing temperature was adjusted based on the specific primers used. Amplified products were electrophoresed on an 8% non-denaturing polyacrylamide gel and stained using the rapid silver staining method (Shang et al., 2009). For the PCR product of *Pid3*, 10 μL was utilized as a template, digested with the restriction enzyme *Bam*HI (TaKaRa, Dalian, China) at 37°C for 1 hour, followed by agarose gel electrophoresis for detection.

**Table 1:** Molecular markers used in this study.

Target genes	Chr.	Marker	Forward primer (F)	Reverse primer (R)	Type of marker	Enzyme	Reference
*Bph2*	12	RM463	TTCCCCTCCTTTTATGGTGC	TGTTCTCCTCAGTCACTGCG	SSR		(Sun et al., 2006)
*Pid1*	2	RM262	CATTCCGTCTCGGCTCAACT	CAGAGCAAGGTGGCTTGC	SSR		(Chen et al., 2004)
*Pid2*	6	RM527	GGCTCGATCTAGAAAATCCG	TTGCACAGGTTGCGATAGAG	SSR		(Chen et al., 2004)
		RM3	ACACTGTAGCGGCCACTG	CCTCCACTGCTCCACATCTT	SSR		
*Pid3*	6	Pid3-dCAPS	TACTACTCATGGAAGCTAGTTCTC	ACGTCACAAATCATTCGCTC	dCAPS	*Bam*HI	(Shang et al., 2009)
*Wx^b^*	6	RM190	CTTTGTCTATCTCAAGACAC	TGCAGATTCTTCCTGATA	SSR		(Bligh et al., 1995)
*Pi5*	9	M-Pi5	ATAGATCATGCGCCCTCTTG	TCATACCCCATTCGGTCATT	Indel		(Gao et al., 2010)
*Pita*	12	RM7102	TTGAGAGCGTTTTTAGGATG	TCGGTTTACTTGGTTACTCG	SSR		(Fjellstrom et al., 2004)

SSR, simple sequence repeat; InDel, insertion and deletion.

### 2.6 Evaluation of key agronomic traits

Throughout the breeding process, observations and analyses of plant and leaf morphology, agronomic traits, and the growth period of rice were conducted to evaluate and select superior individual plants and lines. GH5501, a newly developed restorer line, along with its parents SH527 and FH838, were planted in plots with three replications, each comprising not less than 200 plants, arranged in a randomized block design for the evaluation of yield and agronomic traits. Field water and fertilizer management followed the Guangxi rice regional trial management method.

For the evaluation of agronomic traits, including plant height, number of tillers per plant, total number of grains per spike, fruit set, 1,000-grain weight, and grain length and width, four plants in the middle of each plot were assessed. Statistical analysis was performed using LSD software. Data on the main agronomic traits of NY5501 and TY7118 were obtained from the 2018–2020 regional trials of the late-maturing rice group in the early season in the south of Guangxi.

### 2.7 Heterosis investigation

The selected restorer lines were crossbred with three male-sterile lines: Nafeng A, Tianfeng A, and Longtepu A. Each combination was planted in plots measuring 15 m^2^, repeated three times, with plots arranged randomly. The plant spacing was set at 20 cm × 23 cm. Thirty days after rice heading, measurements of plant height, effective panicles, spikelet number per panicle, 1,000-grain weight, and grain-setting rate were conducted to assess yield traits. The collected data were entered into Excel (Microsoft Office 2003) for analysis, and yield comparisons were made for each combination in the plots. Comprehensive evaluations, considering factors such as plant and leaf morphology and growth period, were performed to preliminarily assess and select superior lines.

### 2.8 Rice quality analysis

The rice quality assessment for all test materials and restorer lines included sending samples to the Rice and Product Quality Supervision, Inspection, and Testing Center of the Ministry of Agriculture and Rural Affairs of China for rice quality analysis. Uniform samples of NY5501 and TY7118 were provided by the Guangxi Rice Variety Regional Trial Group and sent to the Agricultural Ministry Rice and Product Quality Supervision, Inspection, and Testing Center (Hangzhou) for analysis and evaluation. This process followed the NY/T593–2013 standards of the Ministry of Agriculture of China for edible rice variety quality.

### 2.9 Functional gene and resistance gene haplotype analysis of GH5501

During the rice tillering stage, fresh leaves were collected, and DNA extraction was carried out using the CTAB method. Subsequently, the high-density rice gene chip GSR40K was employed to analyze the single-nucleotide polymorphisms (SNPs) of 76 functional genes and resistance genes across the entire rice genome. This chip comprised 32,887 SNPs derived from resequencing results of 4,726 cultivated rice varieties worldwide. The samples were sent to Wuhan Greenfafa Institute of Novel Genechip Research and Development Co. Ltd. (Wuhan, China) for testing. The gene chip analysis detection method is detailed in the literature (Chen et al., 2014).

## 3 Results

### 3.1 Breeding of GH5501

**Figure 1:**
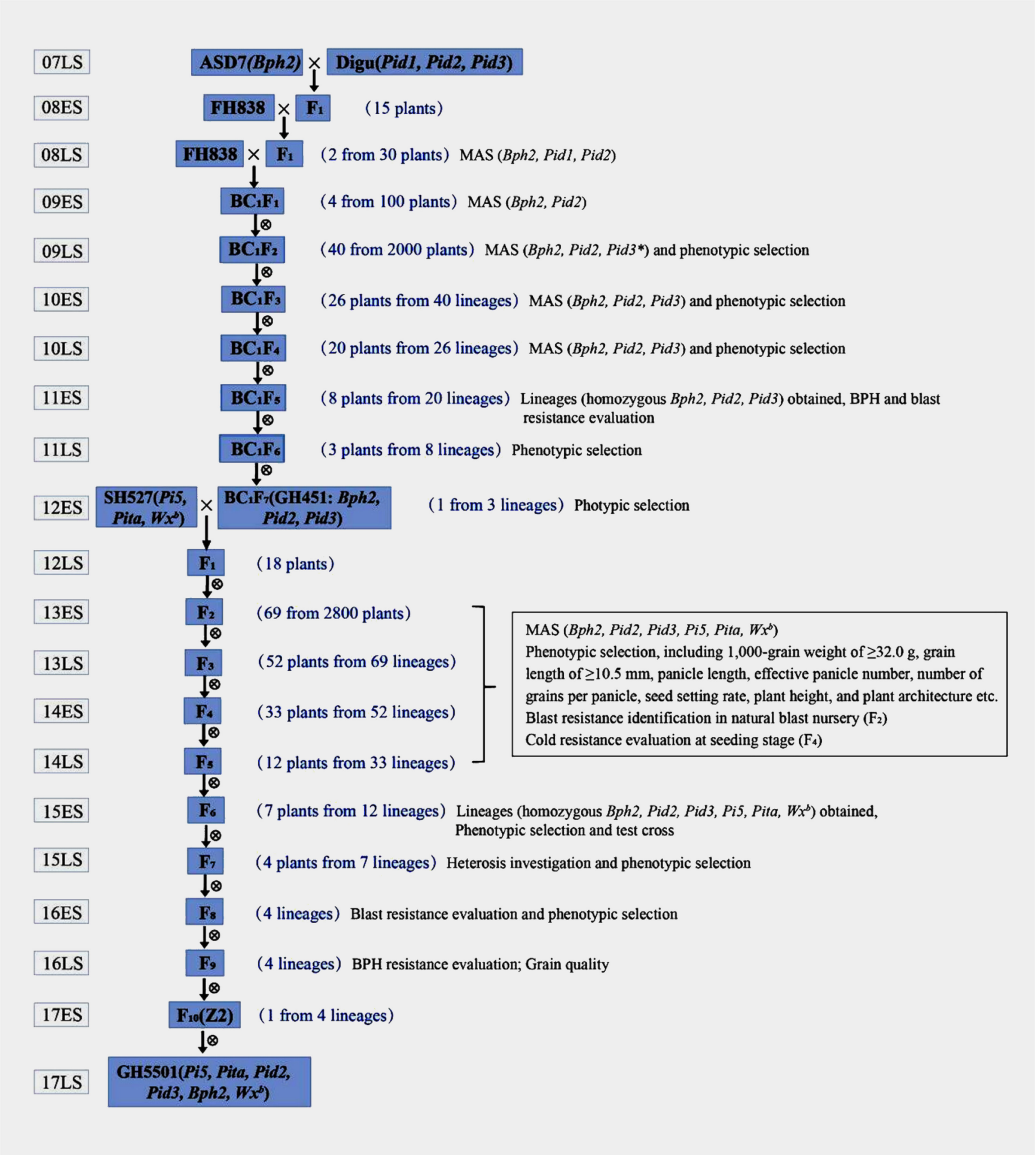
Breeding scheme for the development process of GH5501. “ES” and “LS” indicate the early season and the late season, respectively. In the late season of 2008, molecular markers of *Bph2*, *Pid1*, and *Pid2* were employed to detect three-way cross (FH838//ASD7/Digu) F_1_ plants, two plants of which with both *Bph2* and *Pid2* in a heterozygous state were selected to backcrossed with FH838 (as female parent). BC_1_F_1_ plants were subject to molecular marker detection of *Bph2* and *Pid2* in early season in 2009, *Pid3* gene was published in August 2009, and molecular marker detection of the *Pid3* gene was not incorporated until late season in 2009.

The breeding process of GH5501 was meticulously detailed in **Figure 1**. In the late growing season of 2007, ASD7, a rice variety with intermediate resistance to BPH (containing *Bph2*), was outcrossed as the female parent to Digu, a broad-spectrum blast-resistant germplasm (containing *Pid1*, *Pid2*, and *Pid3*, identified in February 2004 and August 2009, respectively). The resulting F_1_ generation plants were further crossed with the elite heavy-grain restorer line FH838. Two plants from the intercross of FH838//ASD7/Digu, harboring both heterozygous *Bph2* and *Pid2* genes, were selected for backcrossing with FH838 (as the female parent) to produce BC_1_F_1_ plants. Forty plants containing heterozygous *Bph2*, *Pid2*, and *Pid3* genes were selected from BC_1_F_2_ for selfing to produce the BC_1_F_3_ generation in the late season of 2009. The *Pid3* gene was published in August 2009, and molecular marker detection of the *Pid3* gene was not incorporated until the late season of 2009. Subsequently, consecutive self-pollination, MAS, and phenotype-based screening of agronomic traits, particularly heavy grains, were carried out. In the BC_1_F_7_ generation, a line with all three target genes (*Bph2*, *Pid2*, and *Pid3*) in a homozygous state and a 1,000-grain weight of ≥32.0 g was selected and named Guihui451 (GH451) in the early season of 2012.

**Figure 2:**
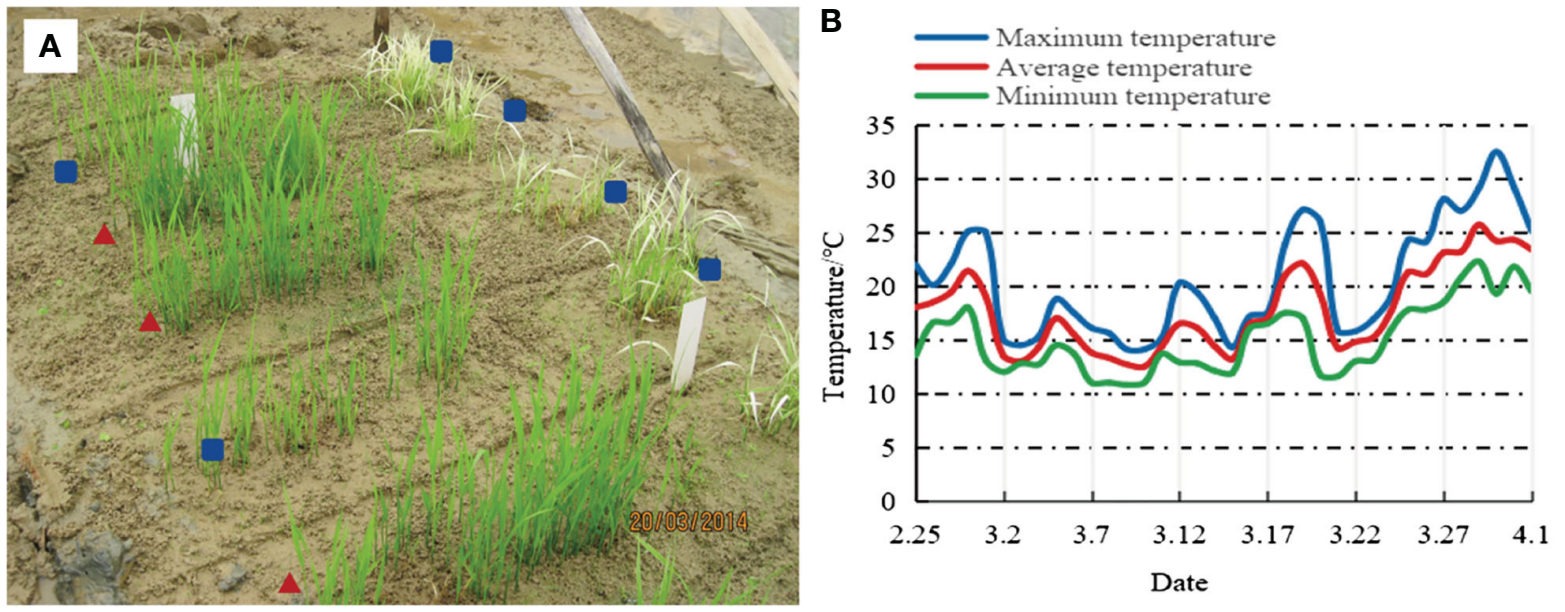
Natural cold tolerance identification in the F_4_ generation. **(A)** Resistance performance, where ▴ indicates selected lineages and ▪ indicates eliminated lineages. **(B)** Temperature fluctuations in Nanning City from February 25 to April 1, 2014.

To further enhance the blast resistance and grain quality of GH451, another elite heavy-grain restorer, SH527 (containing *Pi5*, *Pita*, and *Wx^b^*), was introduced as the male parent for hybridization in the early season of 2012. A total of 2,800 seedlings from the F_2_ generation were grown in a natural blast nursery, and 69 individuals showing high resistance to blast were selected to propagate the F_3_ generation. Starting from the F_3_ to F_6_ generations, MAS was employed in each generation for the six target genes (*Bph2*, *Pi5*, *Pita*, *Pid2*, *Pid3*, and *Wx^b^*). In addition to MAS, phenotypic selection criteria including a 1,000-grain weight of ≥32.0 g, grain length of ≥10.5 mm, panicle length, effective panicle number, number of grains per panicle, seed setting rate, plant height, and plant architecture were applied in our breeding procedure to ensure that selected plants carried the targeted traits. Furthermore, in the F_4_ generation, under low-temperature conditions in the early season of 2014, field selection was conducted for natural cold tolerance. Only individuals showing robust growth, green leaves, and without seedling rot or leaf whitening were chosen for further selection (**Figure 2**). After multiple rounds of selection, seven individuals with homozygous *Bph2*, *Pi5*, *Pita*, *Pid2*, *Pid3*, and *Wx^b^* genes, exhibiting excellent agronomic traits, were carefully selected in the F_6_ generation and test-crossed with three elite CMS lines to evaluate heterosis (**Supplementary Table S1**). Three lines were eliminated, leaving four lines with strong heterosis for further blast, BPH resistance, and grain quality evaluation in the F_8_ and F_9_ generations. After a comprehensive evaluation of all the target traits, an individual line carrying high and broad-spectrum blast resistance, intermediate BPH resistance (rated ≤grade 5.0), strong cold tolerance, heavy grains, and good grain quality were obtained and referred to as Z2. Eventually, after further propagation and selection, the genetically stable Z2 line was produced. Later, the Z2 line was renamed GH5501.

### 3.2 Analysis of functional genes and resistance gene haplotypes in GH5501

A thorough analysis of GH5501 was undertaken using a high-density 40K rice gene chip to scrutinize 76 functional genes and resistance gene alleles. This analysis revealed that GH5501 not only possessed the six target genes identified through molecular markers but also accumulated 20 exceptional alleles (**Table 2**). The results underscored that GH5501 possessed a robust genetic foundation, contributing significantly to its outstanding field performance.

As shown in **Table 2**, eight genes associated with resistance to biotic stressors conferred protection against rice blast, rice yellow mottle virus, rice stripe virus, and BPH. Furthermore, six quality-related genes contributed to the development of heavier and longer grains, accompanied by characteristics such as high protein content, low amylose content, and a higher gelatinization temperature. Four genes related to abiotic stress provided GH5501 with salt tolerance, cold tolerance, and a strong nitrogen absorption capacity. Two high-yield genes and three plant architecture genes contributed to higher yields and a semi-dwarf plant stature with inherent resistance to lodging. GH5501 also carried two heading date genes, one fertility gene, and one lodging-related gene, all closely associated with yield, quality, and resistance. The effective combination of these genes successfully achieved the breeding goal of high yield, quality, and robust stress resistance.

**Table 2:** List of the identified functional genes in GH5501.

No.	Gene	Type	Chr.	Phenotype
1	*Gn1a*	Yield	1	Increased grains per spike
2	*OsSPL16*	Yield	8	High yield
3	*SKC1*	Anti-abiotic stress	1	Salt tolerance
4	*Cold1*	Anti-abiotic stress	4	Cold tolerance
5	*NRT1.1B*	Anti-abiotic stress	10	Increase nitrogen absorption
6	*Rymv1*	Anti-biotic stress	4	Resistance to yellow mottle virus disease
7	*STV11*	Anti-biotic stress	11	Resistance to rice stripe virus
8	*Bph2/Bph18*	Anti-biotic stress	12	Resistance to BPH
9	*Pi5*	Anti-biotic stress	9	Resistance to rice blast
10	*Pia*	Anti-biotic stress	11	Resistance to rice blast
11	*Pid2*	Anti-biotic stress	6	Resistance to rice blast
12	*Pid3*	Anti-biotic stress	6	Resistance to rice blast
13	*Pita*	Anti-biotic stress	12	Resistance to rice blast
14	*OsAAP6*	Quality	1	High protein
15	*GW2*	Quality	2	Large grain
16	*GS3*	Quality	3	Long grain, thermo-tolerance
17	*OsCYP704A3*	Quality	4	Long grain
18	*Wx^b^*	Quality	6	In case of non-waxy, increase the content of amylopectin
19	*ALK*	Quality	6	Increase the content of medium-long amylopectin and gelatinization temperature
20	*Os01g62780*	Heading date	1	Delayed heading
21	*Hd3a*	Heading date	6	Photoperiod sensitivity
22	*S5*	Fertility	6	Wide-compatibility
23	*Sdt97*	Fertility	6	Semi-dwarf
24	*qNGR9*	Plant type	9	Erect panicle
25	*TAC1*	Plant type	9	Increased tillering angle
26	*sh4*	Other type	4	Non-seed shattering

BPH, brown planthopper.

### 3.3 Major agronomic traits and yield performance of GH5501 and its derived hybrid

**Figure 3:**
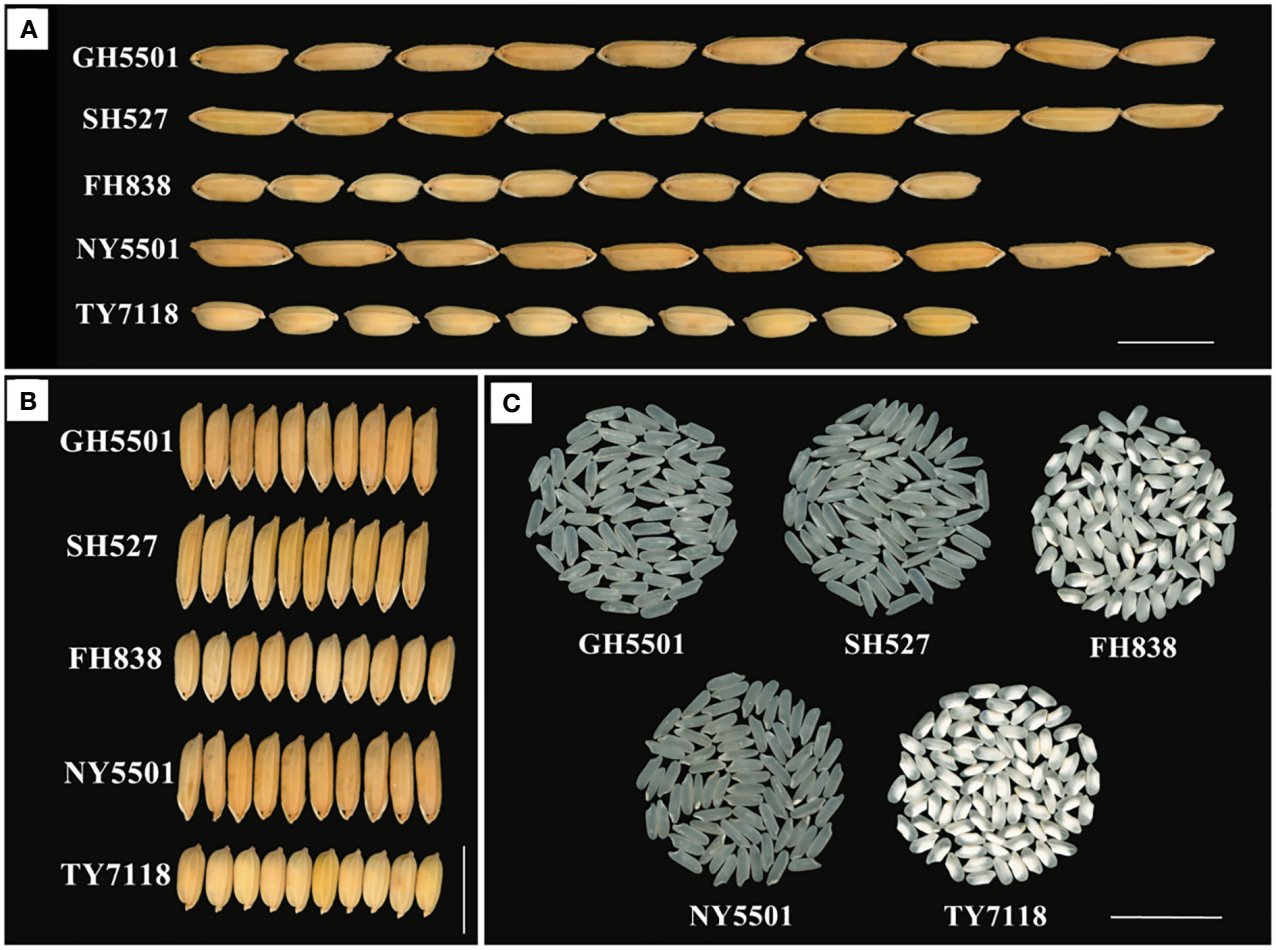
Grain type and rice quality of GH5501 and its derived hybrid NY5501. **(A)** Grain length of GH5501 and its derived hybrid NY5501. Bars = 1 cm. **(B)** Grain width of GH5501 and its derived hybrid NY5501. Bars = 1 cm. **(C)** Rice quality of GH5501 and its derived hybrid NY5501. Bars = 2 cm.

The main agronomic traits of GH5501 are detailed in **Table 3**; **Figure 3**. GH5501 featured a plant height of 116.40 cm, green leaf sheaths, and upright flag leaves. The grain length extended to 10.67 mm, with a grain width of 3.07 mm, resulting in a grain length-to-width ratio of 3.48. The grains were awnless, and the grain type was similar to SH527, but GH5501 had a higher 1,000-grain weight of 35.63 g. It demonstrated a higher effective panicle number, total grain number per panicle, and grain setting rate compared to SH527 and FH838. The differences between GH5501 and SH527 or FH838 in effective panicle number and total grain number per panicle were statistically significant. In addition to its excellent agronomic traits, GH5501 also exhibited prominent characteristics favorable for hybrid seed production. It featured densely packed panicles with abundant pollen. The anthers were well-developed, and the pollen shedding rate exceeded 80%. GH5501 showed great potential in hybrid rice production. In our previous heterosis testing, we observed that the GH5501-Nafeng A (NY5501) hybrid displayed robust hybrid vigor and higher grain quality compared to other testing hybrids we generated.

**Table 3:** Yields and agronomic performances of GH5501 and its derived hybrid under field conditions.

Variety	PH (cm)	NTP	NGP	SSR (%)	GL (mm)	GW (mm)	GLWR	TWG (g)
GH5501	116.40 ± 1.35 a	9.67 ± 0.58 a	239.67 ± 7.51 a	87.91 ± 0.43 a	10.67 ± 0.15 a	3.07 ± 0.15 ab	3.48 ± 0.14 b	35.63 ± 0.47 a
SH527	118.33 ± 1.58 a	7.67 ± 0.58 b	181.33 ± 5.86 b	86.04 ± 0.40 ab	11.13 ± 0.12 a	2.87 ± 0.12 b	3.89 ± 0.14 a	32.90 ± 0.36 b
FH838	110.30 ± 1.74 b	7.33 ± 0.58 b	160.00 ± 6.56 c	84.62 ± 1.59 b	8.97 ± 0.42 b	3.27 ± 0.15 a	2.74 ± 0.01 c	29.83 ± 0.55 c
NY5501	120.07 ± 1.55 a	7.33 ± 0.58 a	169.00 ± 10.00 a	94.46 ± 0.46 a	10.63 ± 0.06 a	3.10 ± 0.10 a	3.43 ± 0.13 a	32.53 ± 0.55 a
TY7118 (CK)	122.53 ± 2.60 a	7.67 ± 0.58 a	158.40 ± 5.82 a	86.76 ± 0.61 b	8.77 ± 0.25 b	3.23 ± 0.15 a	2.71 ± 0.19 b	28.10 ± 1.11 b

Different lowercase letters in the same column indicate significant differences at the 0.05 level using the LSD method. GH5501, FH838, and SH527 were compared with each other, while NY5501 was compared with TY7118.

PH, plant height; NTP, number of tillers per plant; NGP, number of grains/panicle; SSR, seed setting rate; GL, grain length; GW, grain width; GLWR, ratio of length to width; TWG, 1,000-grain weight.

In 2018, the NY5501 hybrid entered the official variety approval trials process in the Guangxi region. That year, its average yield across five test locations was 8,696.22 kg/hm^2^, marking an 8.74% increase over the control variety TY7118 (CK), an elite high-yield benchmark hybrid designated as the control variety for the late-maturing group of the early growing season by the Guangxi rice variety approval committee. Subsequently, in 2019 and 2020, the average yields across six test locations were 8,574.03 kg/hm^2^ and 8,476.35 kg/hm^2^, respectively, representing increases of 5.33% and 6.38% over TY7118 (**Table 4**).

**Table 4:** Yield performances of hybrid combination NY5501 in seven regions of Guangxi.

Year	Variety	Yield in seven regions of Guangxi (kg/hm^2^)							Average yield (kg/hm^2^)	Compared with CK (%)
		Tengxian	Yuling	Wutang	Hepu	Tianyang	Guigang	Qinzhou		
2018	NY5501	8,265.00 ± 121.58 **	8,758.05 ± 200.43 *	9,703.50 ± 237.04**	8,202.00 ± 101.07 **	8,552.55 ± 104.93 **	–	–	8,696.22	8.74
	TY7118 (CK)	7,569.00 ± 102.72	8,265.00 ± 113.74	8,664.00 ± 116.71	7,689.00 ± 117.33	7,799.40 ± 132.29	–	–	7,997.28	
2019	NY5501	8,432.55 ± 126.92 **	8,297.55 ± 68.55 *	7,612.50 ± 161.21	–	9,469.05 ± 55.16 **	9,867.45 ± 332.80 *	7,765.05 ± 53.98 *	8,574.03	5.33
	TY7118 (CK)	7,927.50 ± 113.61	8,074.95 ± 50.28	7,415.70 ± 147.30	–	8,903.40 ± 128.99	9,184.95 ± 143.18	7,332.45 ± 198.68	8,139.83	
2020	NY5501	8,737.50 ± 138.27 **	7,833.45 ± 214.41	8,034.75 ± 135.69	–	9,522.45 ± 249.40 *	9,082.50 ± 251.05 *	7,647.45 ± 115.75 *	8,476.35	6.38
	TY7118 (CK)	8,067.45 ± 115.75	7,442.70 ± 217.14	7,833.75 ± 150.02	–	8,877.45 ± 163.34	8,410.05 ± 89.70	7,174.95 ± 160.16	7,967.73	

From 2018 to 2020, hybrid combinations NY5501 participated in the joint comparative and regional trials for late-maturing rice group of early growing season in south of Guangxi, which included seven different cities and counties.

* and ** represent significances at the 5% and 1% levels, respectively.

To summarize, over the 3-year regional trials conducted at 5–6 test locations each year, NY5501 consistently outperformed the control TY7118, with an average yield of 8,582.20 kg/hm^2^, reflecting a 6.81% increase over the control. Most test locations exhibited significant improvements. Furthermore, in comparison with the control variety TY7118, the NY5501 hybrid demonstrated reduced plant height, increased grain length, grain width, and 1,000-grain weight, along with higher total grain number per panicle and grain setting rate (**Figures 3A, B**; **Table 3**).

As a result of its promising performance, the NY5501 hybrid successfully passed the regional approval trials in 2021 and received government authorization for commercial production.

### 3.4 Rice quality evaluation of GH5501 and its derived hybrid

The grain appearance and main rice quality parameters of GH5501 are presented in **Figure 3C**; **Table 5**. According to the Chinese Ministry of Agriculture and Rural Affairs standard NY/T 593–2013 “Quality Standards for Edible Rice Varieties”, among the seven main indicators influencing rice quality, GH5501 outperformed SH527 and FH838 in four indicators: brown rice rate, head rice rate, alkali spreading value, and gel consistency. GH5501 showed a similar excellent performance as SH527 in two indicators: transparency degree and amylose content. Only its chalkiness degree was slightly inferior to SH527. Compared to SH527 and FH838, GH5501 exhibited good rice quality, with the exception of chalkiness degree and alkali spreading value, meeting the top-grade rice (first grade) of the national standard for premium quality rice (NY/T 593–2013).

**Table 5:** Rice quality of GH5501, its parents, and derived hybrid.

Rice quality detection index	Variety					Grade category of premium quality		
	GH5501	SH527	FH838	NY5501	TY7118 (CK)	1st grade	2nd grade	3rd grade
Brown rice rate (%)^a^	82.3 (1st grade)	80.8 (2nd grade)	81.0 (1st grade)	79.2 (2nd grade)	81.0 (1st grade)	≥81	≥79	≥77
Head rice rate (%)^a^	63.9 (1st grade)	52.4 (3rd grade)	60 (1st grade)	60.9 (1st grade)	49.1 (other)	≥58	≥55	≥52
Chalkiness degree^a^	2.9 (2nd grade)	2.0 (2nd grade)	3.4 (3rd grade)	0.2 (1st grade)	2.6 (2nd grade)	≤1.0	≤3.0	≤5.0
Transparency degree^a^	1.0 (1st grade)	1.0 (1st grade)	-	2.0 (2nd grade)	3 (other)	≤1	≤2	≤2
Alkali spreading value^a^	4.4 (other)	4 (other)	-	3.7 (other)	5.6 (3rd grade)	≥6.0	≥6.0	≥5.0
Gel consistency (mm)^a^	83 (1st grade)	76 (1st grade)	45 (other)	78 (1st grade)	74 (1st grade)	≥60	≥60	≥50
Amylose content (dry basis) (%)^a^	16.9 (1st grade)	16.9 (1st grade)	22 (3rd grade)	15.1 (1st grade)	26.8 (other)	13.0–18.0	13.0–20.0	13.0–22.0

a Evaluation based on the criteria outlined in NY/T593–2013, the “Quality Standards for Edible Rice Varieties” by the Chinese Ministry of Agriculture and Rural Affairs. 1st grade indicates the highest rice quality, followed by 2nd grade and 3rd grade. Varieties reaching 3rd grade or above are considered premium high-quality edible rice, while others are classified as ordinary edible rice.

In comparison to the control TY7118, the hybrid combination NY5501 formed by GH5501 and NF A had longer grains, belonging to the long-grain rice type. Among the seven main indicators affecting rice quality, NY5501 outperformed TY7118 (CK) in five indicators: head rice rate, chalkiness degree, transparency degree, gel consistency, and amylose content. However, it slightly lagged behind TY7118 (CK) in brown rice rate and alkali spreading value. Overall, NY5501 exhibited superior rice quality compared to TY7118 (CK), with most main quality indicators (except for alkali spreading value) meeting the national standard (NY/T 593–2013) required for top-grade rice (second grade or above) (**Figure 3C**, **Table 5**).

### 3.5 Biotic and abiotic stress resistance evaluation of GH5501 and its derived hybrid

**Figure 4:**
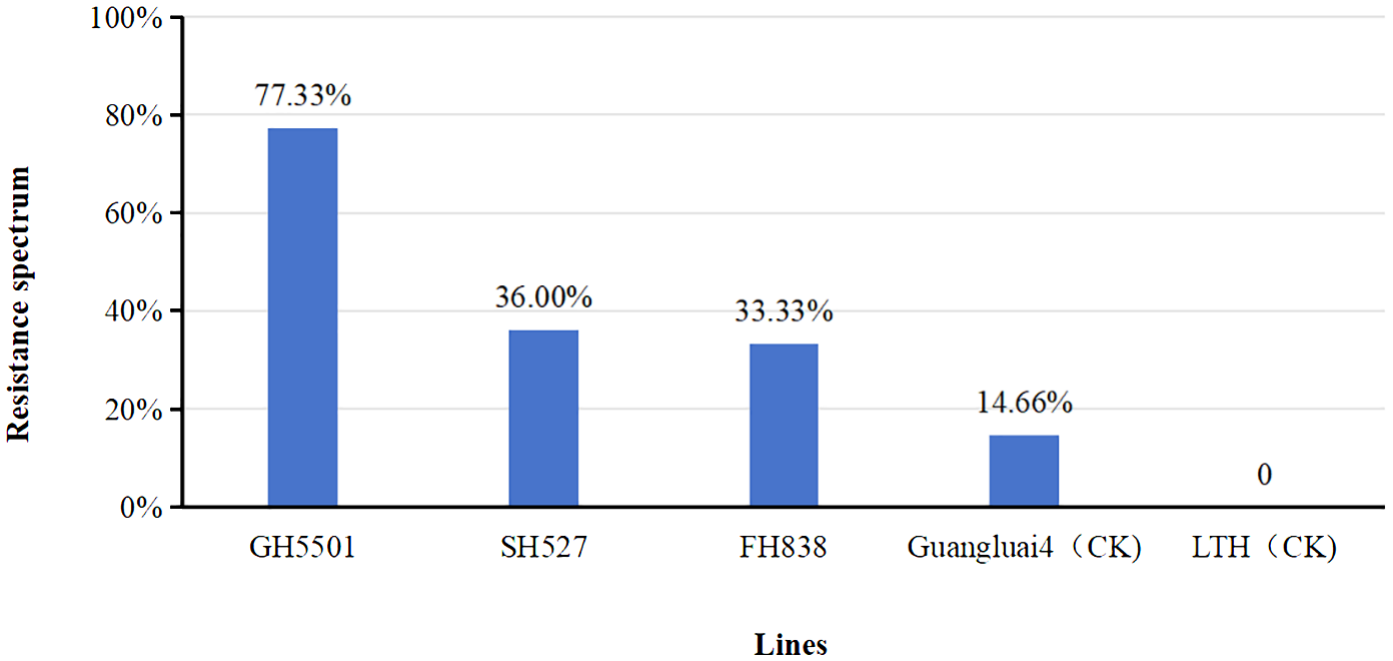
Artificial inoculation of 75 blast isolates for resistance evaluation in GH5501 and heavy-grain parents SH527 and FH838. Abbreviation: LTH, Lijiangxintuanheigu.

To assess the biotic resistance of GH5501, its seedlings were artificially inoculated indoors with 75 isolates of blast fungus collected from various ecological regions in Guangxi and Guangdong provinces for blast disease resistance identification (**Figure 4**, **Supplementary Table S2**). The results revealed that GH5501 exhibited strong resistance to 57 out of the 75 isolates tested, indicating a broad spectrum resistance of 77.32%. In contrast, SH527 and FH838 showed lower resistance to the blast fungus isolates, with resistance frequencies of 36.00% and 33.33%, respectively. Among the two varieties used as controls, Guangluai4 showed resistance to only 14.66% of the isolates, while LTH was susceptible to all 75 isolates tested.

**Figure 5:**
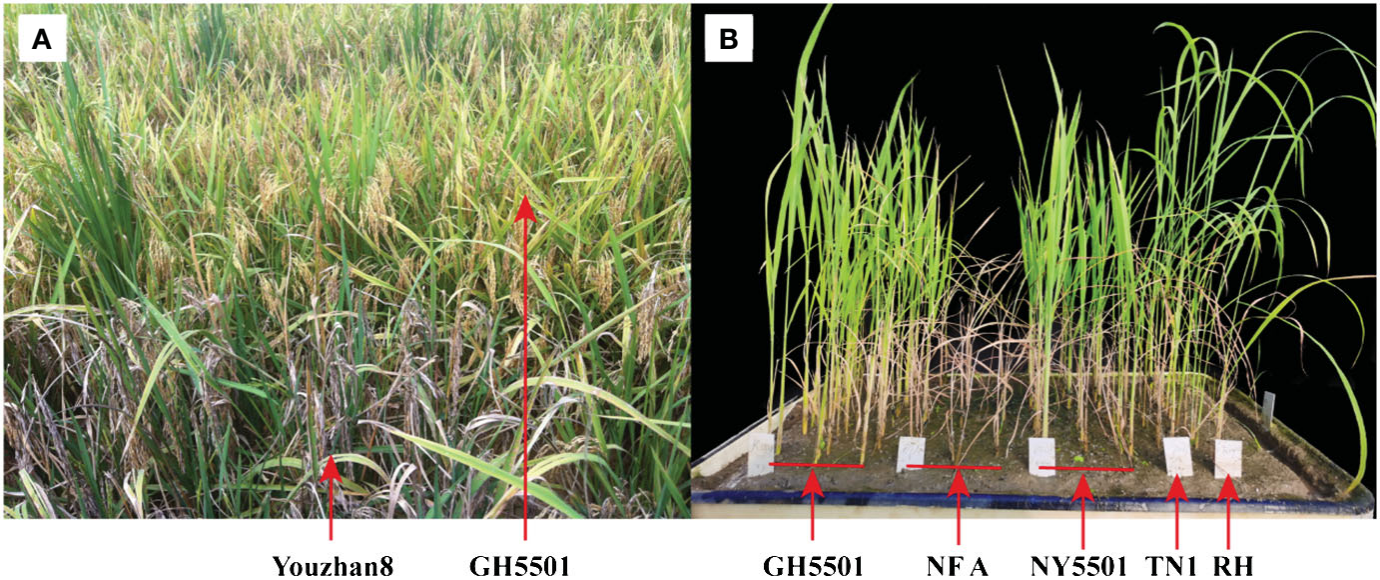
Resistance evaluation of GH5501 and its hybrid combination NY5501. **(A)** Resistance evaluation of GH5501 against blast disease in the natural identification plot, with Youzhan8 as the disease-susceptible control (CK). **(B)** Resistance evaluation of GH5501 and NY5501 against BPH. Rows 1–3 represent GH5501, Rows 4–6 represent NF A, Rows 7–9 represent NY5501, and Rows 10 and 11 represent the susceptible control TN1 and the resistant control RH, respectively. BPH, brown planthopper.

Further investigation into blast resistance under field conditions involved cultivating GH5501 plants in a blast disease evaluation field nursery in Cenxi, Guangxi, where severe blast disease occurred frequently. GH5501 displayed robust high blast resistance, with a leaf blast level of 1.3 and a panicle blast loss rate of 6.1% (level 1.7), indicating overall high resistance (**Figure 5A**; **Table 6**).

**Table 6:** Blast resistance evaluation in GH5501 and its hybrid combination NY5501 under field conditions.

Rice lines	Trait	Level						Resistance level
		Cenxi (2018)	Cenxi (2019)	Hezhou (2019)	Cenxi (2020)	Jinxi (2020)	Average	
GH5501	Leaf blast	1	2	–	1	–	1.3 ± 0.6	R
	Panicle blast loss rate (%)	3.2	11.1	–	4.0	–	6.1 ± 4.3	–
	Panicle blast loss rate (level)	1	3	–	1	–	1.7 ± 0.6	R
NF A	Leaf blast	5	5	–	4	–	4.7 ± 0.6	MS
	Panicle blast loss rate (%)	16.6	23.5	–	21.3	–	20.5 ± 3.5	–
	Panicle blast loss rate (level)	5	5	–	5	–	5.0 ± 0.0	MS
NY5501	Leaf blast	–	4	5	4	3	4.0 ± 0.8 a	MR
	Panicle blast loss rate (%)	–	15.7	5.6	11.6	15.2	12.0 ± 4.7 a	–
	Panicle blast loss rate (level)	–	5	3	3	5	4.0 ± 1.2 a	MR
TY7118 (CK)	Leaf blast	–	5	3	4	3	3.8 ± 1.0 a	MR
	Panicle blast loss rate (%)	–	16.6	3.5	6.6	19.9	11.7 ± 7.8 a	–
	Panicle blast loss rate (level)	–	5	1	3	5	3.5 ± 1.9 a	MR
Youzhan8 (CK)	Leaf blast	7	9	–	9	–	8.3 ± 1.2	S
	Panicle blast loss rate (%)	95.6	98.9	–	95.1	–	96.5 ± 2.1	–
	Panicle blast loss rate (level)	9	9	–	9	–	9.0 ± 0.0	S

The same lowercase letters indicate no significant difference using the LSD method. There are no significant differences between NY5501 and TY7118 at the 0.05 significance level.

R, resistant; MS, moderate susceptibility; MR, moderate resistance; S, susceptible.

NY5501, the hybrid combination of GH5501 and NF A, underwent blast resistance testing for 2 years and four times (twice a year) at three different blast resistance evaluation nurseries. The average leaf blast level of NY5501 was 4.0, and the average panicle blast loss rate was 12.0% (level 4.0), similar to TY7118. Both exhibited moderate resistance to blast disease (**Table 6**), despite NY5501’s maternal line NFA showing intermediate susceptibility to blast.

BPH resistance identification was conducted at the seedling stage on the two parental lines (GH5501 and NFA) and their hybrid NY5501, with RH and TN1 used as resistance and susceptible controls, respectively. The results revealed that GH5501 and NY5501 exhibited resistance levels of 3.78 and 3.93, respectively, indicating moderate resistance to BPH (**Figure 5B**; **Table 7**). Both showed resistance levels similar to the resistance control RH.

**Table 7:** BPH resistance evaluation in GH5501 and its hybrid combination NY5501.

Lines	Average resistant grade	Resistance level
GH5501	3.78 ± 0.18 b	MR
NF A	8.65 ± 0.09 c	S
NY5501	3.93 ± 0.13 b	MR
RH (the resistant control)	3.46 ± 0.13 a	MR
TN1 (the susceptible control)	8.57 ± 0.09 c	S

Letters a to c indicate significantly different values according to statistical analysis using the LSD method (a = 0.05).

MR, moderate resistance; S, susceptible.

Cold tolerance tests were also conducted at the seedling stage on three restorer lines—GH5501, FH838, and SH527—the hybrid NY5501, and IR50 as the susceptible control. The results indicated that GH5501 exhibited a cold tolerance level of 1.80, comparable to that of SH527 (level 1.93), demonstrating strong cold tolerance during the seedling stage. In contrast, FH838 displayed a resistance level of 5.84, a much weaker cold tolerance than GH5501 and SH527, suggesting that the high level of cold resistance in GH5501 was mainly inherited from its parental line SH527. The hybrid combination NY5501 had a resistance level of 3.69, indicating an overall moderate cold tolerance at the seedling stage (**Table 8**).

**Table 8:** Seedling cold tolerance evaluation in GH5501 and its hybrid combination NY5501.

Lines	Average resistant grade	Resistance level
GH5501	1.80 ± 0.08 a	HR
NF A	6.47 ± 0.14 d	MS
NY5501	3.69 ± 0.17 b	MR
SH527	1.93 ± 0.08 a	HR
FH838	5.84 ± 0.12 c	LR
IR50 (CK)	8.73 ± 0.08 e	S

Letters a to e indicate significantly different values according to statistical analysis using the LSD method (a = 0.05).

HR, high resistance; MS, moderate susceptibility; MR, moderate resistance; LR, low resistance; S, susceptible.

## 4 Discussion

China’s southern rice region, located in a tropical and subtropical zone, often encounters prolonged high temperatures during the grain-filling and ripening stages of early rice. This leads to the phenomenon of “heat-induced maturity”, where grains mature quickly before reaching their full size. Consequently, developing high-yielding varieties with heavy grain and good appearance quality becomes challenging. Addressing the trade-off, breeders in this region typically target a 1,000-grain weight of approximately 20.0 g to strike a balance between grain weight and quality, addressing the trade-off (Wang, 2022). While the positive correlation between 1,000-grain weight and yield is well-known, achieving equilibrium between grain weight and quality poses a technical challenge for breeders. In this study, we presented our concerted effort to tackle this challenge. Through a combination of MAS and phenotype-based selection, our team successfully developed GH5501, a high-yielding and good-quality restorer line with long and heavy grains. GH5501 boasted a remarkable 1,000-grain weight of 35.63 g, categorized as an extra-heavy grain variety. Notably, its performance in terms of grain quality, with the exception of alkali spreading value, met or exceeded the standards for high-quality rice (second grade or above) defined by the Chinese Ministry of Agriculture. GH5501 served as a solid foundation for the future breeding of hybrid rice varieties with large and high-quality grains. Varieties with heavy grains are generally favored by farmers due to their characteristics of high and stable yields. The Teyou (TY) hybrid series of rice varieties, derived from CMS Longtepu A, with a 1,000-grain weight generally above 28.0 g, has dominated early rice cultivation in Guangxi, China, since the 1980s. Despite a three-decade-long promotion, these varieties still occupy a significant planting area in Guangxi’s early rice-growing regions (Wang et al., 2015). However, the rice quality of the TY series is generally considered ordinary or poor. The development of the hybrid rice combination NY5501, resulting from the cross between GH5501 and the sterile line NF A, marked a significant achievement. With a 1,000-grain weight of 32.53 g, NY5501 consistently outperformed TY7118 (CK) in yield across 5–6 test points in the late-maturing group in South Guangxi for three consecutive years. The primary grain quality indicators of NY5501 (excluding alkali spreading value) met or exceeded the standards for high-quality rice (level 2 or above), significantly surpassing the control variety (**Table 5**). Considering that the average yield of approved hybrid rice varieties in Guangxi from 2004 to 2018 ranges from 6,910.4 to 8,080.6 kg/hm^2^ (Luo et al., 2022), the yield of NY5501, reaching 8,582.25 kg/hm^2^, indicated its high-yielding and good quality feature. Therefore, GH5501 was deemed an excellent long and heavy grain restorer line. Its utilization held the potential to lead to the development of more high-yielding, good-quality, and stress-tolerant hybrid rice combinations.

Given the escalating occurrence of biotic and abiotic stressors due to frequent climate fluctuations, the development of multi-stress-resistant rice varieties has become imperative to mitigate extensive yield losses (Dixit et al., 2020). Traditional conventional breeding has served as the primary approach for rice breeding since the 1930s. However, achieving the breeding goals of developing varieties resistant to multiple stresses, high-yielding, and good quality through conventional methods has become increasingly challenging (Dixit et al., 2020).

Advancements in rice genome sequencing and molecular biology have facilitated the identification of numerous beneficial genes, leading to significant progress in molecular marker-assisted breeding research (Xiao et al., 2018; Mohapatra et al., 2023). Several successful cases of developing multi-stress-resistant, high-quality, and high-yielding rice varieties have emerged by comprehensively utilizing molecular breeding in conjunction with other breeding methods. For instance, Dixit et al. (2020) successfully aggregated seven to 10 genes/QTLs targeting various biotic and abiotic stressors (e.g., *Pi9* gene for blast; *Xa4*, *xa5*, *xa13*, and *Xa21* genes for bacterial leaf blight; *Bph3* and *Bph17* genes for BPH; *Gm4* and *Gm8* genes for rice gall midge) into the Swarna genetic background using simple sequence repeat (SSR) and gene-based markers, which already has QTLs for drought tolerance (*qDTY1.1* and *qDTY3.1*). This effort resulted in the development of seven introgression lines (ILs) that exhibited higher yields under various stress conditions while maintaining excellent performance under non-stress conditions. Similarly, Mohapatra et al., (2023) successfully introgressed resistance genes (*Xa21*, *xa13*, and *xa5*) for bacterial leaf blight and the QTL *Sub1* for submergence tolerance into the popular Indian late-maturing variety Ranidhan through backcrossing and gene-based markers for MAS. This significantly enhanced its resistance to bacterial leaf blight and submergence tolerance. Liu et al. (2021) and Wang et al. (2022) utilized gene-based markers for MAS and stress screening to breed Huhan 74S (*Pi9*, *Pi5*, and *Pi54*), a two-line sterile line resistant to rice blast, and Huhan 106 (*Pita* and *Pib*), an early-maturing, blast-resistant, and drought-tolerant rice variety. Both varieties have been widely adopted in production. In the current study, a multi-stress-resistant and high-yielding restorer line, GH5501, has been developed through hybridization, multiple crossing, and marker-assisted selection for gene pyramiding (*Pi5*, *Pita*, *Pid2*, *Pid3*, *Bph2*, and *Wx^b^*). We used the linked SSR markers for *Pita*, *Pid2*, *Pid3*, *Bph2*, *Wx^b^*, and gene-based markers for *Pi5* and *Pid3*, but not functional markers for MAS, because our breeding program was started in 2007 when the application of functional markers in rice breeding was not the most efficient and practical method for most of the breeders compared to the SSR and gene-based marker. The prerequisite for the development of functional markers is that the difference in gene function is due to a difference in one or several functional base pairs, which was not successfully developed for every gene. As expected, the integration of MAS with screening for resistance to blast, BPH, and natural cold tolerance, as well as assessments for appearance quality and grain quality, resulted in the successful breeding of GH5501. This restorer line exhibited broad-spectrum resistance to rice blast (with a resistance spectrum of 77.33%), intermediate resistance to BPH, and strong cold tolerance during the seedling stage. The development of GH5501 offered a promising solution for breeding varieties that were not only high-yielding and stress-resistant but also of good quality, contributing to the sustainable improvement of rice production in the face of changing climates.

GH5501 carries four rice blast resistance genes: *Pita*, *Pi5*, *Pid2*, and *Pid3*. *Pita*, located near the centromere on rice chromosome 12, encodes a cytoplasmic membrane receptor protein with a length of 928 amino acids (Bryan et al., 2000). The resistance mediated by *Pita* requires the involvement of *Ptr*, and the NLR protein Pita exhibits broad-spectrum resistance (Zhao et al., 2018; Wang et al., 2021). *Pi5* is situated on rice chromosome 9 and consists of two genes, *Pi5–1* and *Pi5–2*, both producing proteins with CC domains at the N-terminus. Gene expression analysis indicates that *Pi5–1* is pathogen-induced, while *Pi5–2* is consecutively expressed; simultaneous expression of both is necessary for *Pi5*-mediated rice blast resistance. *Pi5* demonstrates broad-spectrum resistance against 26 out of 29 isolates from six isolates in the Philippines and 29 isolates from Korea (Jeon et al., 2003). *Pid2* and *Pid3*, originating from broad-spectrum blast-resistant varieties, are located on rice chromosome 6. *Pid2*, positioned in the centromere-proximal region and linked with RM527 and RM3, encodes a receptor-like kinase, representing a novel type of resistance gene that shows gene-for-gene resistance against the Chinese rice blast strain ZB15 (Chen et al., 2004, Chen et al., 2006; Li et al., 2017). *Pid3*, sharing the same locus as *Pi25*, is on rice chromosome 6 and encodes a 923-amino-acid NBS-LRR domain protein (Shang et al., 2009). These four blast resistance genes have been extensively utilized in breeding for blast resistance, resulting in the development of a range of blast-resistant materials or varieties (Xiao et al., 2018, Xiao et al., 2019; Saichompoo et al., 2021). In this study, we utilized molecular markers to aggregate *Pita*, *Pi5*, *Pid2*, and *Pid3* resistance genes to breed the GH5501 restorer line. Through artificial inoculation with 75 isolates collected from Guangxi and Guangdong, GH5501 exhibited a resistance spectrum of 77.33%. Natural inoculation tests conducted over several years and locations also demonstrated high resistance. These results indicated that the GH5501 restorer line possessed broad-spectrum resistance against rice blast.

Initially, *Bph2* was described as a recessive gene (Athwal et al., 1971). However, Murai et al. challenged this notion, reporting *Bph2* as a dominant gene through genetic analysis of a segregating population of Norin-PL4 crossed with Tsukushibare, a susceptible japonica cultivar (Murai et al., 2001). In the 1970s and 1980s, the International Rice Research Institute developed blast-resistant rice varieties IR36 and IR42 using *Bph2* and extensively promoted them in Southeast Asia (Khush and Brar, 1991). In our breeding program 15 years ago, we utilized ASD7, carrying *Bph2*, as an insect-resistant parent to breed the restorer line GH5501. The hybrid combination NY5501, derived from GH5501, exhibited moderate resistance to the mixed biotype of BPH in the fields of Nanning, Guangxi, demonstrating strong tolerance to BPH. Reports have indicated that varieties developed using *Bph2*, such as the BPH-resistant varieties IR36 and IR42, lose their resistance gradually over 8 years of large-scale promotion due to the emergence of new biotypes of BPH (Sogawa and Kilin, 1987). The Plant Protection Research Team of the Guangxi Academy of Agricultural Sciences, through long-term monitoring of ASD7 (*Bph2*) resistance, shows that its resistance to different biotypes of BPH is unstable, exhibiting moderate resistance to biotype II and moderate susceptibility to the Bangladesh biotype (Wu et al., 2009). In this study, GH5501 bred from the insect-resistant parent ASD7, along with its hybrid combination NY5501, displayed moderate resistance and strong tolerance to BPH, which might be associated with the biotype of BPH. Additionally, this resistance was correlated with the robust growth and stout stems of GH5501. Plant tolerance refers to a plant’s ability to endure or compensate for damage when subjected to the same number of pests as susceptible varieties, relying on its robust growth and reproductive functions (Simms, 2000).

GH5501 exhibited extremely strong cold tolerance during the seedling stage. The F_4_ progeny of this variety endured an average temperature of 17.4°C for 36 days from sowing, and the plants continued to grow robustly with green leaves. No symptoms of white seedlings, damping-off, or dead seedlings were observed (**Figure 2**). Furthermore, cold tolerance assessments confirmed that GH5501 possessed high cold resistance, with a resistance level of 1.80. Gene chip analysis of GH5501 revealed the presence of the cold-tolerance gene *Cold1*, validating its cold tolerance at the molecular level. The *Cold1* gene encodes a G-protein signaling regulator, COLD1, which interacts with the G-protein α-subunit RGA1 to sense low temperatures, activate Ca^2+^ channels, and enhance G-protein GTPase activity, thereby increasing rice cold tolerance (Ma et al., 2015).

Practically, our use of a backcross strategy was avoided in favor of a multiple-crossing method. This decision prevented improvement within the genetic background of a single parent, preserving the genetic diversity of the core parents SH527 and FH838. Simultaneously, we integrated broad-spectrum blast resistance genes and BPH resistance genes from the resistant varieties Digu and ASD7, respectively. Although this extended the breeding cycle, GH5501 selected through this approach showed significant improvements in stress resistance, yield, and quality compared to the parental lines SH527 and FH838 (**Figures 3**, **4**, **Tables 4**, **5**, **8**). Notably, this represented a breakthrough in the breeding of heavy-grain varieties in the Southern rice region, particularly in terms of quality.

Gene chips play an increasingly important role in modern rice breeding, as they enable high-throughput detection of a large number of target genes (Li et al., 2023; Zhang et al., 2023). However, the current high cost of gene chip testing, ranging from USD 70 to 80 per rice sample in China, makes it impractical for large-scale screening of target genes in individual plants from segregating populations. Presently, gene chips are primarily utilized for backcross genetic background analysis and molecular characterization of specific genetic materials, such as parents or varieties (Ren et al., 2021). Gene chip analysis helps to gain a clear understanding of the genetic characteristics of hybrid rice parents, aiding in the rational selection of parent combinations to overcome their shortcomings and create the most hybrid combinations.

Our analysis of GH5501 using gene chips revealed that, in addition to the six target genes selected through molecular MAS, GH5501 also harbored 20 excellent allelic genes, including genes such as *NRT1.1B* for efficient nitrogen utilization, *SKC1* for salt tolerance, and *STV11* for resistance to rice stripe virus. Notably, GH5501 lacked the major-effect gene *Chalk5*, which controls rice appearance quality, milling yield, and the total amount of storage proteins, with a significant impact on various rice quality traits (Li et al., 2014). Therefore, to further enhance the rice quality of future hybrid combinations involving GH5501, selecting hybrid parents with the *Chalk5* gene for compatibility testing with GH5501 is expected. This approach aims to breed hybrid rice varieties with lower chalkiness, thereby further improving rice quality.

The cost of producing hybrid rice seeds has emerged as a major impediment to the widespread adoption of hybrid rice cultivation. Mechanized seed production offers an effective solution to this issue. The diversity in grain types plays a crucial role in the mechanical production of hybrid rice seeds. Chinese breeders have actively explored approaches to tackle this challenge by cultivating small-grain male-sterile lines and heavy-grain restorer lines. They capitalize on substantial differences in grain types, particularly in grain thickness and 1,000-grain weight, as exemplified by varieties such as Zhuoliangyou 2115 and Zhuoliangyou 141 (with a female parent 1,000-grain weight of 14.10 g and male parents at 33.40 g and 28.20 g, respectively). This strategy facilitates the entire process of mechanical seed production, from mixed sowing of parental lines to mechanical separation after harvest, thereby reducing costs and enhancing efficiency in hybrid rice seed production (Lu et al., 2023; Ying et al., 2024). Consequently, the selection of parental lines with significant differences in grain characteristics is pivotal. The long and heavy-grain rice restorer line GH5501, developed in this study with a 1,000-grain weight as high as 35.63 g, exhibited strong heterosis in the resulting hybrid combinations. It could be effectively paired with common small-grain male-sterile lines, creating favorable conditions for mechanized rice seed production. GH5501 emerged as a promising and excellent rice restorer line with potential applications in the field.

## 5 Conclusion

This study successfully employed MAS and conducted multiple resistance screenings to develop GH5501, a novel restorer line characterized by long and heavy grains, low amylose content, broad-spectrum resistance to rice blast, resistance to BPH, and tolerance to low-temperature stress. Using GH5501 as a parent, a new hybrid rice variety named NY5501 was developed, demonstrating high yield, good quality, and resilience to both biotic and abiotic stresses. NY5501 was approved in Guangxi as a rice variety. GH5501 stood as a valuable resource for subsequent three-line hybrid rice breeding, providing a combination of high yield, good quality, and robust resistance to various stressors.

## Data availability statement

The original contributions presented in the study are included in the article/**Supplementary Material**. Further inquiries can be directed to the corresponding authors.

## Author contributions

MW: Writing – original draft, Data curation, Formal Analysis, Investigation, Methodology, Software. QY: Data curation, Formal Analysis, Investigation, Writing – review & editing. DH: Data curation, Formal Analysis, Writing – review & editing, Resources, Supervision. ZM: Data curation, Formal Analysis, Writing – review & editing, Methodology. SC: Data curation, Writing – review & editing, Investigation, Resources. XY: Data curation, Investigation, Writing – review & editing, Formal Analysis, Software. CL: Writing – review & editing, Data curation, Investigation, Methodology, Project administration. YQ: Writing – review & editing, Data curation, Project administration, Software. XZ: Writing – review & editing, Data curation, Investigation, Methodology. ZW: Writing – review & editing, Methodology, Software. YL: Writing – review & editing, Software, Data curation. LY: Writing – review & editing, Software, Formal Analysis. GQ: Writing – review & editing, Conceptualization, Data curation, Project administration, Supervision, Validation. YZ: Writing – review & editing, Conceptualization, Data curation, Supervision, Funding acquisition, Resources, Writing – original draft.

## References

[B1] Andaya V. C. Mackill D. J. (2003). Mapping of QTLs associated with cold tolerance during the vegetative stage in rice. J. Exp. Bot. 54, 2579–2585. doi: 10.1093/jxb/erg243 12966040

[B2] Athwal D. S. Pathak M. D. Bacalangco E. H. Pura C. D. (1971). Genetics of resistance to brown planthopper and green leafhoppers in *Oryza sativa* L. Crop Sci. 11, 747–750. doi: 10.2135/cropsci1971.0011183X001100050043x

[B3] Bligh H. Till R. I. Jones C. A. (1995). A microsatellite sequence closely linked to the *Waxy* gene of *Oryza sativa* . Euphytica 86, 83–85. doi: 10.1007/BF00022012

[B4] Bryan G. T. Wu K. S. Farrall L. Jia Y. Hershey H. P. McAdams S. A.. (2000). A single amino acid difference distinguishes resistant and susceptible alleles of the rice blast resistance gene *Pi-ta* . Plant Cell 12, 2033–2046. doi: 10.2307/3871103 11090207 PMC150156

[B5] Chen X. W. Li S. G. Xu J. C. Zhai W. X. Ling Z. Z. Ma B. T.. (2004). Identification of two blast resistance genes in a rice variety, digu. J. Phytopathol. 152, 77–85. doi: 10.1046/j.1439-0434.2003.00803.x

[B6] Chen X. Shang J. Chen D. Lei C. Zou Y. Zhai W.. (2006). A B-lectin receptor kinase gene conferring rice blast resistance. Plant J. 46, 794–804. doi: 10.1111/j.1365-313X.2006.02739.x 16709195

[B7] Chen H. Xie W. He H. Yu H. H. Chen W. (2014). A high-density SNP genotyping array for rice biology and molecular breeding. Mol. Plant 7, 541–553. doi: 10.1093/mp/sst135 24121292

[B8] Devanna B. N. Jain P. Solanke A. U. Das A. Thakur S. Singh P. K.. (2022). Understanding the dynamics of blast resistance in rice-*magnaporthe oryzae* interactions. J. Fungi 8, 584. doi: 10.3390/jof8060584 PMC9224618 35736067

[B9] Dixit S. Singh U. M. Singh A. K. Alam S. Venkateshwarlu C. Nachimuthu V. V.. (2020). Marker assisted forward breeding to combine multiple biotic-abiotic stress resistance/tolerance in rice. Rice 13, 29. doi: 10.1186/s12284-020-00391-7 32472217 PMC7260318

[B10] Du B. Zhang W. Liu B. Hu J. Wei Z. Shi Z.. (2009). Identifcation and characterization of *Bph14*, a gene conferring resistance to brown planthopper in rice. Proc. Natl. Acad. Sci. U.S.A. 106, 22163–22168. doi: 10.1073/pnas.0912139106 20018701 PMC2793316

[B11] Fjellstrom R. Conaway-Bormans C. A. Mcclung A. M. Marchetti M. A. Shank A. R. Park W. D. (2004). Development of DNA markers suitable for marker assisted selection of three *Pi* genes conferring resistance to multiple Pyricularia grisea pathotypes. Crop Sci. 44, 1790. doi: 10.2135/cropsci2004.1790

[B12] Gao L. J. Gao H. L. Yan Q. Zhou M. Zhou W. Y. Zhang J.. (2010). Establishment of markers for four blast genes and marker distribution in rice parents (in Chinese with English abstract). Hybrid Rice 25, 294–298. doi: 10.16267/j.cnki.1005-3956.2010.s1.090

[B13] Gu S. Zhang Z. Li J. Sun J. Cui Z. Li F.. (2023). Natural variation in OsSEC13 HOMOLOG 1 modulates redox homeostasis to confer cold tolerance in rice. Plant Physiol. 193, 2180–2196. doi: 10.1093/plphys/kiad420 37471276

[B14] Hao J. Wang D. Wu Y. Huang K. Duan P. Li N.. (2021). The GW2-WG1-OsbZIP47 pathway controls grain size and weight in rice. Mol. Plant 14, 1266–1280. doi: 10.1016/j.molp.2021.04.011 33930509

[B15] Huang L. Sreenivasulu N. Liu Q. (2020). *Waxy* editing: old meets new. Trends. Plant Sci. 25, 963–966. doi: 10.1016/j.tplants.2020.07.009 32828690

[B16] International Rice Research Institute(IRRI) (2013). Standard evaluation system (SES) for Rice. 5th edn (IRRI, Manila, the Philippines: IRRI), 46.

[B17] Jantaboon J. Siangliw M. Im-Mark S. Jamboonsri W. Vanavichit A. Toojinda T. (2011). Ideotype breeding for submergence tolerance and cooking quality by marker-assisted selection in rice. Field Crop Res. 123, 206–213. doi: 10.1016/j.fcr.2011.05.001

[B18] Jeon J. S. Chen D. Yi G. H. Wang G. L. Ronald P. C. (2003). Genetic and physical mapping of *Pi5*(*t*) a locus associated with broad-spectrum resistance to rice blast. Mol. Genet. Genomics 269, 280–289. doi: 10.1007/s00438-003-0834-2 12756540

[B19] Khush G. S. Brar D. S. (1991). Genetics of resistance to insects in crop plants. Adv. Agron. 45, 223–274. doi: 10.1016/S0065-2113(08)60042-5

[B20] Li Y. Fan C. Xing Y. Yun P. Luo L. Yan B.. (2014). *Chalk5* encodes a vacuolar H^(+)^-translocating pyrophosphatase influencing grain chalkiness in rice. Nat. Genet. 46, 398–404. doi: 10.1038/ng.2923 24633159

[B21] Li Q. L. Feng Q. Wang H. Q. Kang Y. H. Zhang C. H. Du M.. (2023). Genome-wide dissection of quan 9311A breeding process and application advantages. Rice Sci. 30, 552–566. doi: 10.1016/j.rsci.2023.06.004

[B22] Li S. Gao F. Xie K. Zeng X. Cao Y. Zeng J.. (2016). The OsmiR396c-OsGRF4-OsGIF1 regulatory module determines grain size and yield in rice. Plant Biotechnol. J. 14, 2134–2146. doi: 10.1111/pbi.12569 27107174 PMC5095787

[B23] Li W. Zhu Z. Chern M. Yin J. Yang C. Ran L.. (2017). A natural allele of a transcription factor in rice confers broad-spectrum blast resistance. Cell 170, 114–126.e15. doi: 10.1016/j.cell.2017.06.008 28666113

[B24] Lin L. Wang Y. Xu X. Teng B. Wei C. (2023). Relationships between starch molecular components and eating and cooking qualities of rice using single-segment substitution lines with different *Wx* loci. J. Cereal Sci. 114, 103765. doi: 10.1016/j.jcs.2023.103765

[B25] Liu Y. Zhang F. Luo X. Kong D. Zhang A. Wang F.. (2021). Mol breeding of a novel PTGMS line of WDR for broad-spectrum resistance to blast using *Pi9*, *Pi5*, and *Pi54* genes. Rice 14, 96. doi: 10.1186/s12284-021-00537-1 34825287 PMC8617131

[B26] Lou G. Bhat M. A. Tan X. Wang Y. He Y. (2023). Research progress on the relationship between rice protein content and cooking and eating quality and its influencing factors. Seed Biol. 2, 16. doi: 10.48130/SeedBio-2023-0016

[B27] Lu X. D. Li F. Xiao Y. H. Wang F. Zhang G. L. Deng H. B.. (2023). Grain shape genes: shaping the future of rice breeding. Rice Sci. 30, 379–404. doi: 10.1016/j.rsci.2023.03.014

[B28] Lu G. Wu F. Q. Wu W. Wang H. J. Zheng X. M. Zhang Y.. (2014). Rice *LTG1* is involved in adaptive growth and fitness under low ambient temperature. Plant J. 78, 468–480. doi: 10.1111/tpj.12487 24635058

[B29] Luo T. P. Chen H. W. Qin G. M H. L. (2022). Analysis of characteristics of rice varieties approved in guangxi, China, (2004–2018). Mol. Plant Breed. 1–17.

[B30] Ma Y. Dai X. Xu Y. Luo W. Zheng X. Zeng D.. (2015). *COLD1* confers chilling tolerance in rice. Cell 160, 1209–1221. doi: 10.1016/j.cell.2015.01.046 25728666

[B31] Mao D. Xin Y. Tan Y. Hu X. Bai J. Liu Z. Y.. (2019). Natural variation in the *HAN1* gene confers chilling tolerance in rice and allowed adaptation to a temperate climate. Proc. Natl. Acad. Sci. U.S.A. 116, 3494–3501. doi: 10.1073/pnas.1819769116 30808744 PMC6397538

[B32] Mohapatra S. Barik S. R. Dash P. K. Lenka D. Pradhan K. C. Raj K. R. R.. (2023). Mol breeding for incorporation of submergence tolerance and durable bacterial blight resistance into the popular rice variety ‘Ranidhan’. Biomolecules 13, 198. doi: 10.3390/biom13020198 36830568 PMC9953461

[B33] Murai H. Hashimoto Z. Sharma P. N. Shimizu T. Muratam K. Takumi S.. (2001). Construction of a high-resolution linkage map of a rice brown planthopper (Nilaparvata lugens Stål) resistance gene *bph2* . Theor. Appl. Genet. 103, 526–532. doi: 10.1007/s001220100598

[B34] Murray M. G. Thompson W. F. (1980). Rapid isolation of high molecular weight plant DNA. Nucleic Acids Res. 8, 4321–4325. doi: 10.1093/nar/8.19.4321 7433111 PMC324241

[B35] Ning X. Yun W. Aihong L. (2020). Strategy for use of rice blast resistance genes in rice molecular breeding. Rice Sci. 27, 263–277. doi: 10.1016/j.rsci.2020.05.003

[B36] Pennisi E. (2010). Armed and dangerous. Science 327, 804–805. doi: 10.1126/science.327.5967.804 20150482

[B37] Ren Y. Chen D. Li W. Tao L. Yuan G. Q. Cao Y.. (2021). Genome-wide pedigree analysis of elite rice Shuhui 527 reveals key regions for breeding. J. Integr. Agr. 20, 35–45. doi: 10.1016/S2095-3119(20)63256-7

[B38] Saichompoo U. Narumol P. Nakwilai P. Thongyos P. Malumpong C. (2021). Breeding novel short grain rice for tropical region to combine important agronomical traits, biotic stress resistance and cooking quality in koshihikari background. Rice Sci. 28, 479–492. doi: 10.1016/j.rsci.2021.07.008

[B39] Seck F. Covarrubias-Pazaran G. Gueye T. Bartholome J. (2023). Realized genetic gain in rice: achievements from breeding programs. Rice 16, 61. doi: 10.1186/s12284-023-00677-6 38099942 PMC10724102

[B40] Shang J. Tao Y. Chen X. Zou Y. Lei C. Wang J.. (2009). Identification of a new rice blast resistance gene, *Pid3*, by genomewide comparison of paired nucleotide-binding site–leucine-rich repeat genes and their pseudogene alleles between the two sequenced rice genomes. Genetics 182, 1303–1311. doi: 10.1534/genetics.109.102871 19506306 PMC2728867

[B41] Simms E. L. (2000). Defining tolerance as a norm of reaction (in Chinese with English abstract). Evol. Ecol. 14, 563–570. doi: 10.1023/A:1010956716539

[B42] Sogawa K. Kilin D. (1987). Biotype shift in a brown planthopper(BPH) population on IR42. Int. Rice Res. Newslett. 12, 40.

[B43] Sun L. H. Wang C. M. Changchao S. U. Liu Y. Q. Zhai H. Q. Wan J. M. (2006). Mapping and marker-assisted selection of a brown planthopper resistance gene *bph2* in rice (*Oryza sativa* L.). Acta Genetica Sin. 33, 717–723. doi: 10.1016/S0379-4172(06)60104-2 16939006

[B44] Tacconi G. Baldassarre V. Lanzanova C. Faivre-Rampant O. Cavigiolo S. Urso S.. (2010). Polymorphism analysis of genomic regions associated with broad-spectrum effective blast resistance genes for marker development in rice. Mol. Breed. 26, 595–617. doi: 10.1007/s11032-010-9394-4

[B45] Temnykh S. Park W. D. Ayers N. Cartinhour S. Mccouch S. R. (2000). Mapping and genome organization of microsatellite sequences in rice (*Oryza sativa* L.). Theor. Appl. Genet. 100, 697–712. doi: 10.1007/s001220051342

[B46] Teng B. Zeng R. Wang Y. Liu Z. Zhang Z. Zhu H.. (2012). Detection of allelic variation at the *Wx* locus with single-segment substitution lines in rice (*Oryza sativa* L.). Mol. Breed. 30, 583–595. doi: 10.1007/s11032-011-9647-x

[B47] Teng B. Zhang Y. Wu J. Cong X. Wang R. Han Y.. (2013). Association between allelic variation at the *Waxy* locus and starch physicochemical properties using single-segment substitution lines in rice (*Oryza sativa* L.). Starch Strke 65, 1069–1077. doi: 10.1002/star.v65.11-12

[B48] Wang F. (2022). Breeding and development of high-quality hybrid rice in south China (in Chinese with English abstract). China Rice 28, 107–116. doi: 10.3969/j.issn.1006-8082.2022.05.016

[B49] Wang F. Liu Y. Zhang A. Kong D. Bi J. Liu G.. (2022). Breeding an early maturing, blast resistance water-saving and drought-resistance rice(WDR) cultivar using marker-assisted selection coupled with rapid generation advance. Mol. Breed. 42, 46. doi: 10.1007/s11032-022-01319-3 37313512 PMC10248681

[B50] Wang J. Wang R. Fang H. Zhang C. Zhang F. Hao Z.. (2021). Two VOZ transcription factors link an E3 ligase and an NLR immune receptor to modulate immunity in rice. Mol. Plant 14, 253–266. doi: 10.1016/j.molp.2020.11.005 33186754

[B51] Wang S. J. Wei M. J. Feng Z. G. Huang J. Yan Y. M. Zeng L. (2015). Breeding and application of good-quality *Indica* hybrid rice Teyou 7118 (in Chinese with English abstract). Zhongzi (Seed) 34, 108–110. doi: 10.16590/j.cnki.1001-4705.2015.04.054

[B52] Wu B. Q. Huang F. K. Huang S. S. Long L. P. Wei S. M. (2009). Resistance stability of rice varieties to different biotypes of brown planthopper (in Chinese with English abstract). J. App. Ecol. 20, 1477–1482. Available at: https://www.cjae.net/CN/abstract/abstract2304.shtml. 19795662

[B53] Xiao W. Peng X. Luo L. Liang K. Wang J. Huang M.. (2018). Development of elite restoring lines by integrating blast resistance and low amylose content using MAS. J. Integr. Agr. 17, 16–27. doi: 10.1016/S2095-3119(17)61684-8

[B54] Xiao W. Yang Q. Huang M. Guo T. Liu Y. Wang J.. (2019). Improvement of rice blast resistance by developing monogenic lines, two-gene pyramids and three-gene pyramid through MAS. Rice 12, 78. doi: 10.1186/s12284-019-0336-4 31686256 PMC6828908

[B55] Yan L. Luo T. Huang D. Wei M. Ma Z. Liu C.. (2023). Recent advances in molecular mechanism and breeding utilization of brown planthopper resistance genes in rice: an integrated review. Int. J. Mol. Sci. 24, 12061. doi: 10.3390/ijms241512061 37569437 PMC10419156

[B56] Yang Y. Z. Wang K. Fu C. J. Qin P. Xie Z. M. Hu X. C.. (2020). hybrid rice Analysis on grain quality improvement of Indica hybrid rice in China (in Chinese with English abstract). Hybrid Rice 35, 1–7. doi: 10.16267/j.cnki.1005-3956.20190923.238

[B57] Ying J. Z. Qin Y. B. Zhang F. Y. Duan L. Cheng P. Yin M.. (2024). A weak allele of *TGW5* confers higher seed propagations and efficient size-based seed sorting for hybrid rice production. Plant Commun 5, 100811. doi: 10.1016/j.xplc.2024.100811 38213029 PMC11009153

[B58] Zeng D. Tian Z. Rao Y. Dong G. Yang Y. Huang L.. (2017). Rational design of high-yield and superior-quality rice. Nat. Plants 3, 17031. doi: 10.1038/nplants.2017.31 28319055

[B59] Zhang C. P. Li M. Liang L. P. Xiang J. Zhang F. Zhang C. Y.. (2023). Rice3K56 is a high-quality SNP array for genome-based genetic studies and breeding in rice (*Oryza sativa* L.). Crop J. 11, 800–807. doi: 10.1016/j.cj.2023.02.006

[B60] Zhao H. Wang X. Jia Y. Minkenberg B. Wheatley M. Fan J.. (2018). The rice blast resistance gene *Ptr* encodes an atypical protein required for broad-spectrum disease resistance. Nat. Commun. 9, 2039. doi: 10.1038/s41467-018-04369-4 29795191 PMC5966436

[B61] Zhao J. Zhang S. Dong J. Yang T. Mao X. Liu Q.. (2017). A novel functional gene associated with cold tolerance at the seedling stage in rice. Plant Biotechnol. J. 15, 1141–1148. doi: 10.1111/pbi.12704 28173633 PMC5552475

[B62] Zhu M. Liu Y. Jiao G. Yu J. Zhao R. Lu A.. (2024). The elite eating quality alleles *Wx^b^ * and *ALK^b^ * are regulated by OsDOF18 and coordinately improve head rice yield. Plant Biotechnol. J. 22, 1582–1595. doi: 10.1111/pbi.14288 38245899 PMC11123401

